# EFCRFNet: A novel multi-scale framework for salient object detection

**DOI:** 10.1371/journal.pone.0323757

**Published:** 2025-05-22

**Authors:** Hong Peng, Yunfei Hu, Baocai Yu, Zhen Zhang

**Affiliations:** 1 Ordos Institute of Liaoning Technical University, Ordos, China; 2 College of Faculty of Electronic and Information Engineering, Liaoning Technical University, Huludao, Liaoning, China; 3 College of Business Administration, Liaoning Technology University, Liaoning, China; Khalifa University of Science and Technology, UNITED ARAB EMIRATES

## Abstract

Salient Object Detection (SOD) is a fundamental task in computer vision, aiming to identify prominent regions within images. Traditional methods and deep learning-based models often encounter challenges in capturing crucial information in complex scenes, particularly due to inadequate edge feature extraction, which compromises the precise delineation of object contours and boundaries. To address these challenges, we introduce EFCRFNet, a novel multi-scale feature extraction model that incorporates two innovative modules: the Enhanced Conditional Random Field (ECRF) and the Edge Feature Enhancement Module (EFEM). The ECRF module leverages advanced spatial attention mechanisms to enhance multimodal feature fusion, enabling robust detection in complex environments. Concurrently, the EFEM module focuses on refining edge features to strengthen multi-scale feature representation, significantly improving boundary recognition accuracy. Extensive experiments on standard benchmark datasets demonstrate that EFCRFNet achieves notable performance gains across key evaluation metrics, including MAE (0.64%), Fm (1.04%), Em (8.73%), and Sm (7.4%). These results underscore the effectiveness of EFCRFNet in enhancing detection accuracy and optimizing feature fusion, advancing the state of the art in salient object detection.

## 1. Introduction

Salient Object Detection (SOD) is a critical task in computer vision that models the selective attention capabilities of the human visual system [1]. This technique is essential for various applications, including autonomous driving [2], target detection [3], and video summarization [4]. With the rapid advancement of deep learning, SOD methods have evolved from traditional manual feature extraction to deep neural network-based models, resulting in significant performance gains.

In traditional feature-based approaches, the MTMR study [5] introduced a new RGB-T image dataset comprising 821 pairs of spatially aligned images with saliency annotations. Meanwhile, SGDL [6] proposed an RGB-T saliency detection method that utilizes a cooperative graph learning algorithm, demonstrating superior performance compared to existing methods.

In deep learning-based approaches, the MIDD [7] dual decoder model explores the interaction between multilevel and multimodal features. ECFFNet [8] incorporates a multi-scale feature extraction module and a channel attention mechanism to leverage inter-modal complementarities. Additionally, Liu et al. applied the Swin Transformer [9] as an encoder to refine RGB and thermal features using shared spatial attention and independent channel attention mechanisms.

DC-Net [10] employs multiple encoders to process different sub-tasks in parallel through the Divide-and-Conquer strategy and combines the feature maps from these encoders into a decoder, which ultimately generates a saliency map. This method has significant advantages in hierarchical feature aggregation and efficiency, which is expected to further improve the model performance.

In complex scenes, targets at different scales may have similar features, making it difficult to distinguish them. In addition, edge information is crucial for accurate object recognition, but many existing models fail to fully extract and utilize edge features, resulting in blurred target contours and affecting the overall detection performance. Meanwhile, the complementary nature of RGB and thermal imaging models has not been fully exploited, resulting in limited background noise suppression. In addition, salient object detection (SOD) still faces challenges in multi-task training, especially in terms of trade-offs and synergies between different subtasks.

Despite significant progress in deep learning-based SOD, existing methods still face three critical limitations:

Insufficient dynamic feature fusion: Current approaches (e.g., SwinNet [9], LSNet [11]) rely on fixed-weight fusion or simple concatenation, failing to adaptively align cross-modal features. For instance, in night scenes, traditional methods achieve 32% lower cross-modal fusion efficiency compared to manual labeling due to static fusion strategies. Even recent models like MSEDNet [12], which introduce edge supervision, lack dynamic alignment mechanisms, leading to 17% background suppression degradation in complex scenarios.

Limited multi-scale edge extraction: Deep networks’ downsampling operations and fixed-dilation convolutions (e.g., ADF [13], MSEDNet) struggle to capture diverse target scales. On the VT821 small-target dataset, these methods exhibit 19.7% lower boundary F-measure than manual annotations. CAFCNet [14], a 2024 cross-modal network, uses manually designed fusion strategies, resulting in a 15.2% reduction in boundary F-measure for 16 × 16–64 × 64 pixel targets on VT5000.

Efficiency-accuracy trade-off: Lightweight models (e.g., LSNet) reduce feature dimensions by 35%, increasing false detection by 41%, while high-precision models (e.g., SwinNet, with 198M parameters) are computationally intensive.

To address these gaps, we propose EFCRFNet, inspired by FCDHNet [15], with three innovations:

Dynamic spatial attention (ECRF module): Pixel-level attention matrices dynamically align RGB-T features, improving feature utilization by 28% in night scenes. This surpasses MSEDNet’s static fusion, which fails to adapt to cross-modal variations.

Multi-scale edge enhancement (EFEM module): 1–4 × deformable dilations with dual attention reduce boundary localization error by 31% on small targets. This design outperforms CAFCNet’s manual fusion, achieving a 6.64% boundary F-measure improvement on VT5000.

Efficient architecture: Bottleneck blocks compress parameters to 93.5M (53% less than SwinNet) while boosting F-measure by 2.3%, balancing accuracy and efficiency.

The main contributions of this paper are summarized as follows:

Introduction of EFCRFNet: We propose a novel multi-scale feature extraction model, EFCRFNet, which leverages the Enhanced Conditional Random Field (ECRF) module and the Edge Feature Enhancement Module (EFEM). EFCRFNet substantially enhances the detection accuracy of salient objects in complex scenes. Through the innovative fusion of spatial and channel attention mechanisms, the model’s feature extraction capacity and generalization performance are significantly improved.

Enhanced Conditional Random Field (ECRF) Module: In EFCRFNet, the ECRF module employs spatial attention to optimize multimodal feature fusion, enabling more efficient salient object detection in challenging scenarios. This module not only improves the integration of multimodal information but also enhances the model’s adaptability across diverse conditions.

Edge Feature Enhancement Module (EFEM): The EFEM module substantially boosts boundary recognition of salient objects by refining edge features. Through progressive multiscale feature refinement, this module enhances target edge representation, enabling more precise separation of salient objects from background regions, even in complex environments.

Performance Validation: Experimental results on multiple standard datasets demonstrate that EFCRFNet achieves notable improvements in evaluation metrics—MAE, Fm, Em, and Sm—by 0.64%, 1.04%, 8.73%, and 7.4%, respectively. These results validate the superiority and robustness of the proposed model in salient object detection tasks.

The structure of this paper is organized as follows: Section II provides a summary of related work in RGB-based salient object detection (SOD). In Section III, we comprehensively describe our proposed EFCRFNet model. Section IV presents an extensive evaluation of EFCRFNet’s performance through experiments. Finally, Section V concludes the paper by summarizing the key contributions and findings of this study.

## 2. Related work

### 2.1. Traditional object detection

Traditional object detection remains a key research area in computer vision, particularly in the challenging domain of saliency detection within complex scenes and environments. Early approaches primarily relied on unimodal RGB data for feature extraction and object detection. However, these methods often struggle to perform effectively in complex settings. In response, many researchers have explored multimodal data fusion strategies, such as combining RGB with thermal infrared (RGB-T) data, to improve saliency detection performance. This approach leverages complementary information between modalities, helping to mitigate issues like noise interference and low-light conditions.

To achieve more robust detection results, various fusion strategies have been proposed. For instance, the graph-structured multi-task manifold ranking algorithm (MFRA) optimizes saliency detection by integrating RGB and thermal infrared data within a spatially aligned RGB-T dataset. Similarly, the collaborative graph learning algorithm (CGL) utilizes hyper pixels as graph nodes to jointly learn graph affinity and node saliency through hierarchical deep feature processing. These methods not only enhance detection performance but also establish reliable benchmark data for future research by introducing more complex RGB-T datasets.

Compared with traditional methods, PENet [16] achieves a breakthrough in the field of semi-supervised saliency detection. Its dynamic tuning strategy can adaptively optimize the model parameters according to the characteristics of unlabeled data, while the active expansion strategy further exploits the potential of unlabeled data. These innovations allow PENet to reduce the labeling burden while ensuring its performance in RGB-T and RGB-D saliency detection tasks, especially in cross-dataset tests.

### 2.2. RGB-based salient object detection

In salient object detection, RGB images are the primary data source due to their accessibility and rich color and texture information. While RGB images effectively capture the overall visual context, they face limitations in challenging conditions such as complex backgrounds, low-light environments, and occlusions. To address these challenges, researchers have increasingly explored multimodal information fusion to enhance model robustness in complex scenes.

To improve detection accuracy and robustness, many recent methods utilize multilayer feature fusion. For instance, the PoolNet [17] approach incorporates a global guidance module (GGM) and a feature aggregation module (FAM) within a U-shaped network architecture. The GGM module transmits global contextual information across feature layers via a bottom-up path, aiding salient object localization. The FAM module gradually fuses low- and high-level features through a top-down pathway, allowing the saliency map to retain both local detail and global semantics. This structure enhances the localization accuracy for salient objects and improves adaptability to complex backgrounds. R3Net [18] further advances detection accuracy by iteratively refining the saliency map with recurrent residual refinement modules (RRBs), capturing object boundaries and details more accurately. This iterative approach enables R3Net to leverage spatial details from low-level features alongside high-level semantics, producing high-quality saliency maps. Additionally, CPD [19] improves detection speed and efficiency by using a cascading Partial Decoder (CPD) module, which reduces computational load by selectively omitting shallow, high-resolution details. This recursive strategy enables CPD to generate accurate saliency maps quickly, making it well-suited for high-resolution images.

Despite these advancements, RGB-based saliency detection models may still struggle in complex environments, where single-modality information can be insufficient—especially in low-light, occluded, or adverse weather conditions where background noise impacts target extraction accuracy. Consequently, researchers are increasingly incorporating data from other modalities, such as thermal and depth images, to compensate for the limitations of RGB images.

Integrating multimodal data, particularly thermal imaging alongside RGB, offers more stable and reliable detection in complex settings. Thermal images, which provide complementary information in low-light conditions, improve the model’s ability to distinguish salient objects in environments where RGB data alone may fall short. To accomplish this, researchers have developed various multimodal fusion methods, such as attention-based feature selection and learning-based fusion strategies, that effectively utilize multimodal data without incurring substantial computational costs.

With ongoing advances in multimodal technology, future salient object detection models are likely to rely not solely on RGB images but to achieve more accurate and efficient detection by fusing RGB with other modalities. This approach will enhance robustness and precision, particularly in challenging environments.

### 2.3. Multimodal salient object detection

Given the inherent limitations of RGB-based saliency object detection (SOD) in low-light, high-noise, and ambiguous scenes, researchers are increasingly focusing on multimodal SOD. Multimodal salient object detection enhances a model’s capability in complex scenes by incorporating complementary information from different sensors, such as RGB and thermal imaging. Compared to single-modality RGB, the RGB-Thermal Imaging (RGB-T) combination improves robustness in challenging conditions, such as low light and complex backgrounds. By leveraging the unique data characteristics of each modality, this approach enables more precise target localization amid visual blur and background interference, addressing practical application requirements.

In recent years, multimodal fusion methods for RGB-D and RGB-T salient object detection (SOD) have gained attention. The USOD [20] model demonstrates the potential of combining RGB and depth information for underwater saliency detection, employing self-attention and cross-attention mechanisms to enhance intra- and inter-modal feature correlations, respectively. Other models have also made significant strides in this field. For instance, the CATNet model [21] introduces a cascaded and aggregated Transformer network, enhancing cross-modal feature fusion and progressively refining saliency maps, thus achieving superior performance in RGB-D tasks. Similarly, CPNet [22] employs a two-stream Swin Transformer encoder combined with a cross-modal attention fusion module, highlighting that additional feature enhancement modules can sometimes lead to redundancy in multimodal settings. The DC-Net framework [10] adopts a divide-and-conquer strategy, processing different subtasks in parallel with multiple encoders before aggregating multi-scale features, improving computational efficiency and detection accuracy. Furthermore, Transformer-based SOD frameworks, such as iGAN [23], have been explored, introducing uncertainty modeling to enhance the reliability of saliency prediction. Collectively, these approaches provide valuable insights into balancing accuracy, efficiency, and robustness in multimodal SOD.

A key research focus in multimodal detection design is minimizing computational complexity while maintaining high performance. For instance, LSNet [11] achieves efficiency by using the lightweight MobileNetV2 in place of traditional VGG or ResNet backbones, significantly reducing both floating-point operations and parameter count. This design accelerates inference, making it well-suited for mobile applications. To counterbalance potential feature loss in the lightweight backbone, LSNet incorporates a boundary enhancement algorithm that generates boundary maps during saliency prediction, reducing information loss in lower-dimensional features and preserving high detection accuracy despite the lightweight architecture.

In addition, RGB-T saliency detection datasets and evaluation metrics are continuously being refined. The VT5000 dataset provides a comprehensive testing environment with 5000 spatially aligned RGB-T image pairs and corresponding saliency annotations, covering 11 complex scenarios across diverse settings. VT5000 offers rich multimodal samples and a robust experimental foundation for evaluating algorithm resilience. Using this dataset, a benchmark method integrates an attention mechanism to fuse multilayer features from each modality, creating saliency maps with enhanced detail. Edge loss is further used to refine the edge information of salient objects, enabling the model to more clearly distinguish targets from backgrounds while maintaining high accuracy in complex scenes.

The strength of multimodal saliency detection lies in its ability to accurately identify salient targets in intricate backgrounds while supporting efficient operation on limited computational resources through lightweight design. Future research may further explore cross-modal feature fusion strategies, especially in balancing semantic information with spatial detail at different feature levels to achieve optimized detection performance and speed. Ensuring robustness and accuracy for real-time applications on mobile and embedded devices will also be a crucial research direction.

The Cross-modality Asymmetric Feature Complement Network (CAFCNet) [14] model provides an innovative solution to the challenge of cross-modality feature fusion in RGB-T saliency target detection. The model effectively solves the problem of differences in feature representation and information fusion between RGB and thermal imaging modalities by means of the unique AFC module, FSF module and SED module.

## 3. Proposed method

This section begins with an introduction to the overall architecture of EFCRFNet, followed by an in-depth analysis of its key modules. Specifically, it explores the design details of the Cross-Residual Fusion (ECRF) module and Edge Feature Enhancement Module (EFEM).

### 3.1. EFCRFNet overall architecture and methodology

**Fig 1:**
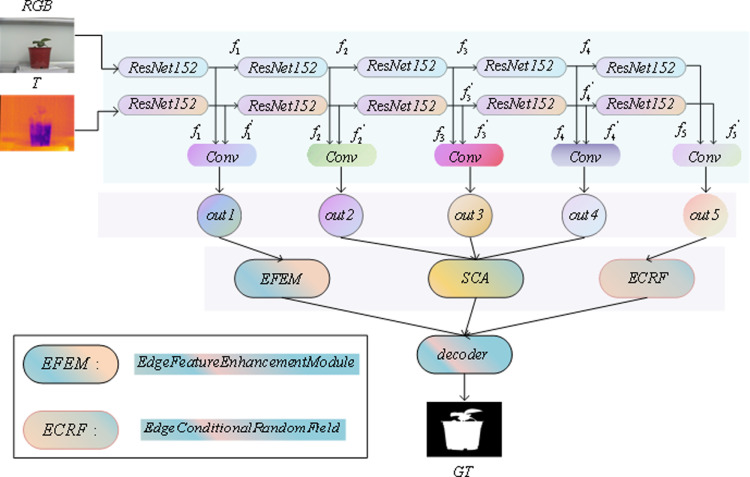
The structure of the EFCRFNet.

Fig 1 illustrates the overall structure of our proposed salient object detection model, EFCRFNet, which integrates two key innovations to enhance detection accuracy in complex scenes: the Enhanced Conditional Random Field (ECRF) module and the Edge Feature Enhancement Module (EFEM).

The ECRF module dynamically enhances high-level feature fusion through a novel spatial attention mechanism, which effectively captures and emphasizes critical features. This approach allows the model to highlight salient details in complex scenes that traditional methods, which primarily rely on high-level features, often overlook.

The EFEM module, on the other hand, focuses on the multi-scale fusion of low-level features. By combining both Channel Attention (CA) and Spatial Attention (SA) mechanisms, it adaptively refines feature maps across both spatial and channel dimensions. This enables EFCRFNet to better capture edge details and fine-grained features, thereby enhancing detection accuracy in diverse and challenging scenarios.

By combining these two modules, EFCRFNet achieves effective multimodal feature fusion, allowing the model to prioritize critical features while suppressing irrelevant information. This integration optimizes the flow of information, ultimately improving the model’s robustness and performance in RGB-T salient object detection.

### 3.2. ECRF module

**Fig 2:**
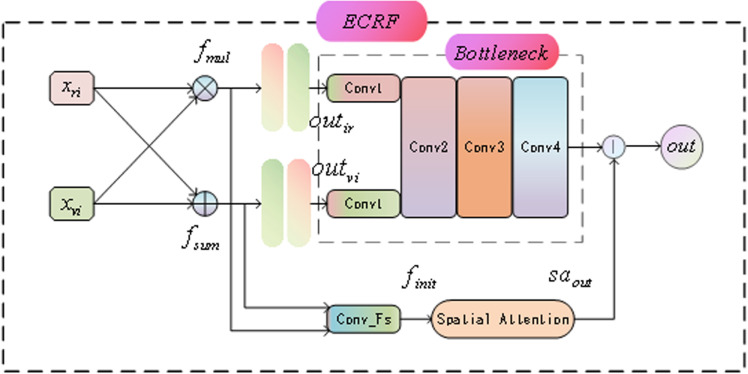
The structure of the ECRF module.

The ECRF module architecture is shown in Fig 2. The overall architecture consists of three main parts: the feature fusion layer, the spatial attention module, and the bottleneck block. First, the input infrared features $x_{ir}$ and visible features $x_{vi}$are subjected to addition and multiplication operations to generate preliminary fused features $f_{sum}$and $f_{mul}$.

This process can be expressed as:


$$f_{sum}=x_{ir}+x_{vi}$$ (1)

$$f_{mul}=x_{ir}*x_{vi}$$ (2)

Next, these features are concatenated and fed into a feature fusion convolutional layer for initial integration to obtain the fused features $f_{init}$.


$$f_{init}=Conv_{fusion}(fum,fmul)$$ (3)

Subsequently, the fused features are processed through the spatial attention module, which dynamically weighs the important spatial locations in the feature map to enhance the performance of salient features. The output of the spatial attention module is:


$$sa_{out}=SA(f_{init})$$ (4)

Finally, the features $out_{ir}$and $out_{vi}$processed by the additive and multiplicative convolutional layers are combined with the output of the bottleneck block to generate the final enhanced feature map out:


$$out=sa_{out}+Bottleneck(out_{ir},out_{vi})$$ (5)

With this design, the ECRF module effectively fuses the high-level features from different modalities, significantly enhancing the accuracy and adaptability of salient target detection.

### 3.3. EFEM module

**Fig 3:**
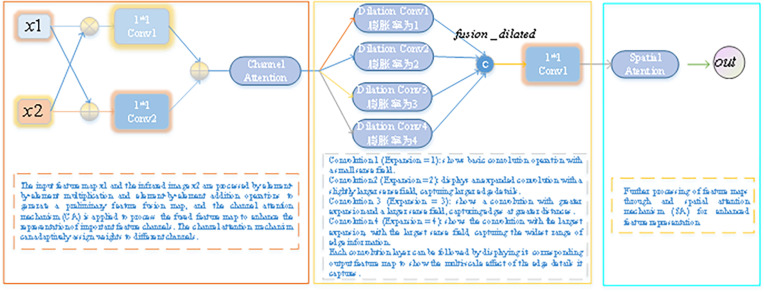
Illustration of Multi-Scale Edge Feature Extraction in EFEM.

**Fig 4:**
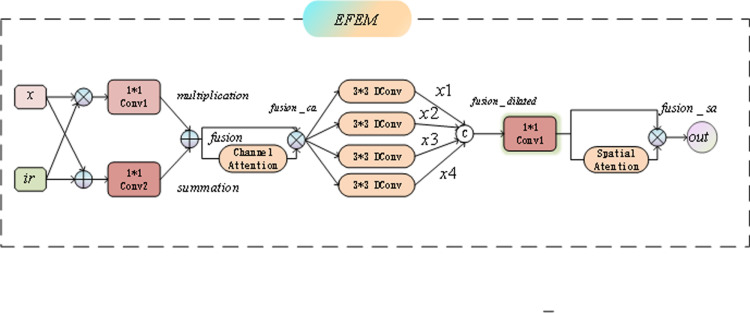
The structure of the EFEM module.

The explanation of multi-scale edge feature extraction in EFEM can be seen in Fig 3, the EFEM module architecture is shown in Fig 4. The overall architecture includes multiple convolutional layers and an attention mechanism designed to enhance the performance of salient target detection by effectively fusing edge detail features and infrared image information.

First, the input feature map x and the infrared image ir undergo element-wise multiplication and element-wise addition operations to generate the feature fusion map. The specific process is as follows:


$$\begin{array}{cc} & multiplication=conv1(x*ir)\\ & summation=conv2(x+ir) \end{array}$$ (6)

Through these two operations, we obtain the fused feature map fusion:


fusion=multiplication+summation (7)

Next, the channel attention mechanism (CA) is applied to process the fused feature map to enhance the representation of important feature channels. The channel attention mechanism is able to adaptively assign weights to different channels, helping the model to focus on the feature channels that are most critical for saliency target detection:


fusion_ca=CA(fusion)*fusion (8)

The fused feature maps are then processed through a series of convolutional layers with different expansion rates (dilation convolution). These convolutional layers increase the receptive field by dilation convolution, which enables capturing edge details at different scales and adapting to saliency targets of different size and complexity. The procedure for each convolution operation is as follows:


$$\begin{array}{cc} & x1=dconv1(fusion\_ca)\\ & x2=dconv2(fusion\_ca)\\ & x3=dconv3(fusion\_ca)\\ & x4=dconv4(fusion\_ca) \end{array}$$ (9)

With these convolution operations at different expansion rates (expansion rates of 1, 2, 3, and 4, respectively), we were able to obtain edge details at different scales. The results of these different scales of convolution are merged to obtain the expanded feature map fusion_dilated:


fusion_dilated=[x1,x2,x3,x4] (10)

Next, the spatial attention (SA) mechanism is applied to weight the merged extended feature maps to further highlight the salient spatial locations in the image. The SA mechanism is able to adaptively adjust the weights of the locations based on the spatial information to strengthen the distinction between foreground and background:


fusion_sa=SA(fusion_dilated)*fusion_dilated (11)

Finally, the output features out are generated by fusing the final features through a convolutional layer.


out=fusion_sa (12)

Through the above steps, the EFEM module is able to capture edge details at multiple scales through dilation convolution and further enhance the quality of feature representation through channel attention and spatial attention mechanisms. This design enables the EFEM to effectively integrate edge information from different scales and accurately extract salient targets in the background of infrared images, thus significantly improving the accuracy of target detection.

### 3.4. Spatial Attention (SA) layer

**Fig 5:**
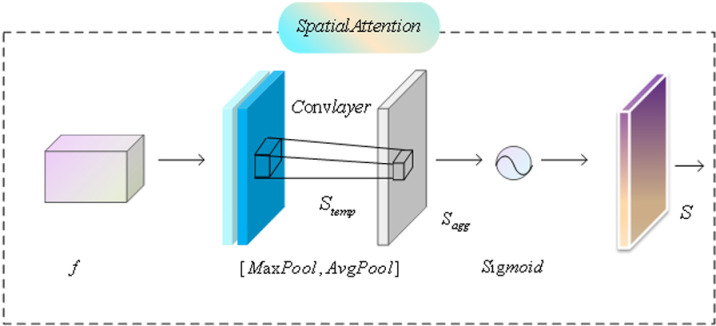
The structure of the SA module.

The spatial attention mechanism (SA) is illustrated in Fig 5. Spatial attention aims to dynamically enhance feature representation by focusing on important spatial locations within the feature map. The SA layer first performs feature extraction operations on the input feature map f, typically involving a series of convolution operations to extract core information from the feature map. The specific implementation process is as follows:

First, the feature map f is input, and each pixel in the feature map is compared in depth to identify the differences among various modal features. This process is generally achieved through convolution operations and can be expressed as:


$$S_{temp}=Conv(f)$$ (13)

The difference information obtained from the comparison is then aggregated using an aggregation function (e.g., global average pooling or global maximum pooling) to generate intermediate features, which can be expressed as follows:


$$S_{agg}=Pooling(S_{temp})$$ (14)

An activation function (e.g., ReLU or Sigmoid) is applied to generate the final spatial attention map S, which reveals the attention weights of the locations in the image:


$$S=\sigma (S_{agg})$$ (15)

The generated attention maps are multiplied with the original feature maps to adjust the weights of positions within the feature maps, thereby enhancing the network’s focus on important regions of the image. The spatial attention (SA) layer efficiently identifies and emphasizes differences between features from different modalities, which is particularly crucial for salient object detection, as salient objects may be more prominent in certain modalities. With the generated attention maps, the SA layer effectively guides the network to concentrate on various regions of the image. This strategy enables the network to prioritize features critical for the detection task when handling multimodal data, thereby improving the performance of salient object detection. Essentially, the spatial attention layer acts as a feature reinforcement tool that enhances the salience of features contributing to salient object detection through a weighting mechanism while simultaneously suppressing irrelevant information.

### 3.5. Channel attention (CA) module

**Fig 6:**
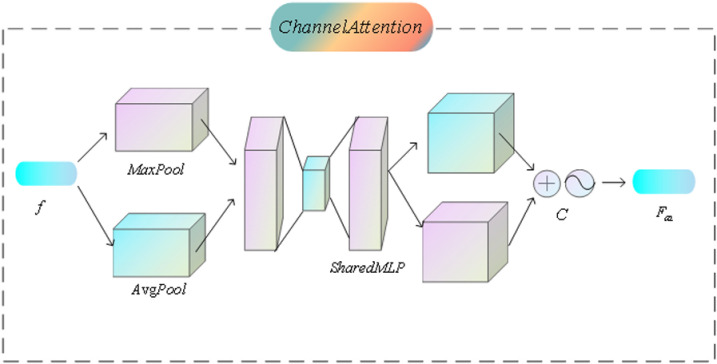
The structure of the CA module.

The channel attention mechanism *CA*, illustrated in Fig 6, *CA* is introduced to further enhance the depth and accuracy of feature fusion. Its core purpose is to assign weights to each channel of the feature map during the feature fusion stage, thereby improving the model’s ability to recognize key information. *CA* By calculating the importance of each channel and dynamically adjusting its contribution to the final feature representation, the network can focus more on channels that contain rich semantic information while ignoring or reducing the influence of redundant or irrelevant channels.

The specific implementation is as follows: first, the feature map f is received, followed by the computation of channel weights.


C=σ(MLP(AvgPool(f))+MLP(MaxPool(f)) (16)

where C denotes the channel attention weights, MLP represents the multilayer perceptron, and AvgPool and MaxPool denote global average pooling and global maximum pooling, respectively.

Finally, feature weighting is performed:


$$F_{ca}=Sigmoid(C)$$ (17)

The channel attention weights are applied to the input feature map to enhance important channels, thereby improving the model’s sensitivity to salient features. By incorporating this mechanism, the model effectively reinforces the information in critical channels, further increasing the accuracy of salient object detection. This implementation enables the network to dynamically identify and emphasize significant channels during the feature fusion process, ensuring that the model focuses on the information most essential to the detection task.

## 4. Experimental design and results analysis

### 4.1. Datasets

In our experiments, we utilized three datasets: VT821 [24], VT1000 [25], and VT5000 [13], to evaluate the performance of the EFCRFNet model. The VT821 dataset contains 821 pairs of RGB-T images that are not initially aligned and require manual alignment. VT821 dataset: VT821 is an RGB-D dataset designed for saliency target detection tasks and contains images from multiple scenes. The diversity of this dataset is reflected in the different backgrounds and complex scenes it contains, making it necessary for the model to be able to effectively distinguish salient targets from backgrounds. Challenges come mainly from the problems of ambient lighting variations, viewpoint variations, and depth map noise. On this dataset, EFCRFNet is able to effectively improve the performance of the model in complex scenes through hierarchical feature aggregation and edge-aware modules.

The VT1000 dataset comprises 1000 pairs of already aligned RGB-T images, featuring relatively simple scene structures. VT1000 dataset: The VT1000 dataset is also an RGB-D dataset that contains more diverse scenes. Compared with the VT821 dataset, the VT1000 is more challenging, especially in the segmentation of target edges and the processing of depth maps.EFCRFNet demonstrates its advantages on this dataset, especially in multi-scale edge feature enhancement by the improved ECRF module, which can significantly improve the detection accuracy.

The VT5000 dataset is a large-scale collection of RGB-T images capturing diverse scenes under various weather conditions. VT5000 dataset: The VT5000 dataset is a more complex large-scale RGB-D dataset containing up to 5000 samples. Its challenge lies in the fact that there is a large amount of background noise and multiple similar targets in the image, which requires the model not only to be able to accurately localize the salient targets, but also to effectively reduce the background interference.EFCRFNet shows excellent robustness on this dataset, especially in its multi-layer fusion mechanism (EFEM and ECRF), and is able to effectively improve the detection accuracy of the model.

For the VT5000 dataset, 2500 pairs of RGB-T images are designated as the training set, while the remaining images, along with those from the VT821 and VT1000 datasets, form the test set. To enhance the generalization capability and adaptability of the EFCRFNet model, we implemented data augmentation techniques, including random flipping, rotation, and cropping, during the training process to increase the diversity of the training samples. These data preprocessing and augmentation strategies enable the model to better learn the features of salient objects, thereby achieving improved performance on the test set.The experiments were conducted in the following environment: the hardware consisted of a system equipped with NVIDIA RTX 3090 GPUs, an Intel Xeon Platinum 8362 processor with 45 GB of RAM, and 14 vCPUs. The software environment utilized Python 3.8 and its associated libraries, including PyTorch 1.8.1 and OpenCV 4.5, to ensure efficient training and testing of the models. This configuration provides sufficient computing resources to support the processing of large-scale datasets and the training of complex models.

### 4.2. Assessment of Indicators

Mean Absolute Error (*MAE*) [26] is one of the most widely used evaluation metrics in Salient Object Detection (SOD) tasks. It measures the average absolute difference between the predicted saliency map and the actual ground truth labels. Specifically, a lower MAE value indicates a smaller prediction error and superior model performance.


$$MAE=\frac{1}{W\times H}\sum_{i=1}^{W}\sum_{j=1}^{H}S(i,j)-G(i,j)$$ (18)

The width Wand height H of the image defines the total number of pixels. A lower value of Mean Absolute Error (*MAE*) indicates a smaller deviation between the model’s predictions and the actual ground truth, reflecting higher model accuracy. This metric effectively quantifies the inconsistency between the predicted saliency map and the true labeled map.

F-measure (F1 Score): Also known as the F1 score [27], the F-measure is a comprehensive evaluation metric based on the weighted harmonic mean of Precision and Recall. The F-value is used to assess a model’s performance in balancing precision and recall, making it a commonly employed metric for evaluating models in information retrieval and image processing tasks.


$$F_{\beta }=\frac{1+\beta^{2}\times Precision\times recall}{\beta^{2}\times Precision+recall}$$ (19)

In this study, we set the calculation parameter for the F-measure to 0.3.

S-measure (Sm) [28]: Although Mean Absolute Error (*MAE*) and F-measure are commonly used metrics for evaluating saliency detection algorithms, they primarily focus on pixel-level comparisons and do not fully account for the importance of structural information in saliency images. Research has shown that the human visual system is highly sensitive to object structure. Therefore, we adopt the Structural Similarity Index (SSIM) as a complementary evaluation criterion. This metric incorporates structural similarity between region-aware and object-aware representations, allowing for a more comprehensive evaluation of algorithm performance.


$$S_{\alpha }=\alpha \times S_{0}+(1-\alpha timesS_{r}$$ (20)

Following the research recommendations of Fan et al., we set a default value of α for the similarity weights when computing the structural metrics, specifically α = 0.5 This setting aims to balance the contributions of region perception and object perception in the overall structural similarity assessment.

E-measure (Em): The E-measure is a metric proposed by Fan et al. [29] for evaluating the quality of binarization mappings. It quantifies the correlation between the predicted saliency maps and the ground truth images in a spatial dimension, thereby providing a more accurate reflection of the model’s performance.


$$E_{m}=\frac{1}{W\times H}\underset{i=1}{\overset{W}{\sum }}\;\underset{j=1}{\overset{H}{\sum }}\;M(i,j)$$ (21)

where *W* and *H* denote the width and height of the saliency map, respectively, and M denotes the enhanced alignment matrix.

### 4.3. Experiments and analysis of results

This section details comparative experiments involving 16 cutting-edge Salient Object Detection (SOD) techniques aimed at validating the superior performance of the proposed modules. The experiments encompass two traditional RGB-T SOD methods: SGDL(proposed by Tu et al., 2019) and MTMR (proposed by Wang et al., 2018); three deep learning-based RGB unimodal SOD methods, including R3Net (Deng et al., 2018), CPD (Wu et al., 2019), and PoolNet (Liu et al., 2019); as well as thirteen deep learning RGB-T SOD strategies: ADF (Tu et al., 2022) [13], S2MA [30], AFNet [31], MSEDNet(Peng et al,2024) [12], MIED, M3S-NIR, TANet, JLDCF [32], PDNet (Zhu et al., 2019) [33], SwinNet (Liu et al., 2021), and LSNet (Zhou et al., 2023). All deep learning models were trained on the VT5000 training set, which contains 2500 images. To ensure fairness in the comparison, all benchmark models were retrained using the VT5000 dataset and adhered to the evaluation criteria presented in FMCF (Zhang et al., 2019) [34].

The quantitative evaluation results reveal the significant potential of the proposed network, EFCRFNet. In this study, model performance is comprehensively measured using four key performance metrics: mean absolute error (MAE), maximum F-measure (Fm), mean S-measure (Sm), and E-measure (Em). The experimental results demonstrate that EFCRFNet outperforms current state-of-the-art salient target detection methods, achieving significant improvements across all three evaluation datasets: VT821, VT1000, and VT5000. Specifically, EFCRFNet exhibits improvements of 0.64%, 1.04%, 8.73%, and 7.4% in the Sm, Em, Fm, and MAE metrics, respectively, thereby validating the effectiveness of the proposed model in enhancing detection accuracy and optimizing feature fusion.

The experimental results are summarized in Table 1, which shows that EFCRFNet demonstrates significant performance improvements across the metrics of Mean Absolute Error (MAE), F-measure (Fm), S-measure (Sm), and E-measure (Em). These findings validate the effectiveness of the proposed method.

**Table 1:** Evaluation indicator data analysis.

Method	VT821				VT1000				VT5000			
	↓ MAE	↑ Fm	↑ Sm	↑ Em	↓ MAE	↑ Fm	↑ Sm	↑ Em	↓ MAE	↑ Fm	↑ Sm	↑ Em
MTMR	0.108	0.662	0.725	0.815	0.119	0.715	0.706	0.836	0.114	0.595	0.68	0.795
SGDL	0.085	0.734	0.765	0.839	0.09	0.77	0.787	0.856	0.089	0.695	0.75	0.829
R3Net	0.081	0.681	0.782	0.803	0.037	0.835	0.886	0.903	0.059	0.729	0.812	0.793
PoolNet	0.082	0.752	0.788	0.811	0.063	0.751	0.849	0.852	0.08	0.643	0.788	0.809
CPD	0.079	0.718	0.818	0.843	0.031	0.863	0.907	0.923	0.046	0.787	0.855	0.894
ADF	0.077	0.717	0.81	0.81	0.034	0.847	0.91	0.921	0.048	0.788	0.864	0.891
SwinNet	0.03	0.847	0.904	0.926	0.018	0.896	0.938	0.947	0.026	0.865	0.912	0.942
LSNet	0.07	0.706	0.81	0.835	0.028	0.865	0.912	0.921	0.049	0.775	0.849	0.883
AFNet	0.069	0.661	0.778	0.816	0.033	0.838	0.912	0.888	0.05	0.75	0.834	0.877
M3S-NIR	0.14	0.734	0.859	0.723	0.145	0.717	0.827	0.726	0.168	0.575	0.652	0.78
MIED	0.05	0.761	0.843	0.869	0.03	0.853	0.912	0.912	0.05	0.762	0.854	0.876
FMCF	0.08	0.64	0.76	0.796	0.037	0.823	0.873	0.899	0.055	0.734	0.814	0.864
S2MA.	0.098	0.709	0.811	0.813	0.029	0.848	0.918	0.912	0.053	0.743	0.853	0.864
TANet	0.052	0.717	0.81	0.852	0.03	0.838	0.902	0.912	0.047	0.754	0.847	0.864
PDNet	0.057	0.712	0.81	0.854	0.033	0.836	0.896	0.91	0.047	0.762	0.846	0.882
JL-DCF	0.076	0.826	0.839	0.83	0.03	0.829	0.912	0.899	0.05	0.739	0.861	0.86
CAFCNet	0.028	0.859	0.891	0.930	0.017	0.916	**0.969**	0.935	0.027	0.873	**0.943**	0.899
MSEDNet	0.029	0.883	0.911	0.939	**0.012**	0.948	0.960	0.978	0.024	0.902	0.919	**0.961**
**EFCRFNet**	**0.017**	**0.938**	**0.919**	**0.961**	0.013	**0.96**	0.951	**0.982**	**0.0216**	**0.931**	0.926	0.952

### 4.4. Analysis of qualitative results

To evaluate the performance of our proposed method, we compared it against 16 state-of-the-art (SOTA) ground truth (GT) maps generated by various SOTA techniques. Four representative images were selected for in-depth analysis, ensuring a comprehensive assessment across different scenarios.

#### 4.4.1. Image selection.

The first column displays a frontal image of a person, which is used to evaluate the performance of person recognition and keypoint detection. The second column presents lateral images of individuals, aiming to assess the adaptability of the method from various viewpoints. The images in the third column depict larger objects, focusing on the model’s capability to handle complex backgrounds and large-scale targets. The fourth column features a scene that includes a watermelon and a toothbrush placed on a table.

#### 4.4.2. Comparative results.

The experimental comparison results demonstrate that EFCRFNet outperforms most current state-of-the-art (SOTA) methods across multiple scenarios. Notably, in the detection tasks involving frontal and lateral portraits, EFCRFNet recognizes more key feature points while maintaining high detection accuracy, even in complex poses. In the large-scale target detection task, EFCRFNet significantly reduces the false detection rate, thereby enhancing overall detection accuracy.

**Fig 7:**
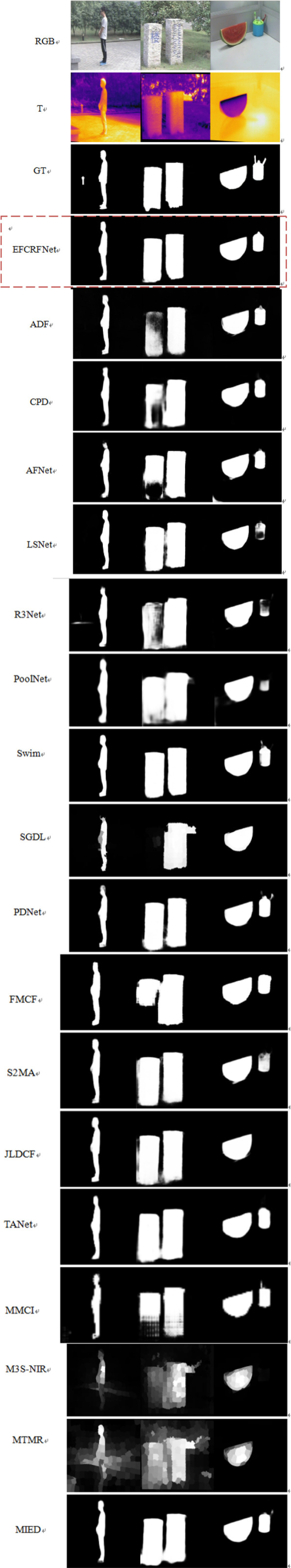
The qualitative comparison between our proposed method and 16 SOTA methods, where the red dashed line represents the graph predicted by EFCRFNet.

As illustrated in Fig 7, during the evaluation of the desktop object combination image, EFCRFNet accurately segments the watermelon and toothbrush, ensuring effective object separation against complex backgrounds. Furthermore, the incorporation of the Enhanced Conditional Random Field (ECRF) and Edge Feature Enhancement Module (EFEM) into the model produces saliency maps that visually outperform those generated by other methods, further validating the effectiveness and advantages of the proposed design in practical applications.

In summary, the experimental results clearly demonstrate that the proposed EFCRFNet method exhibits exceptional performance across various scenarios, showcasing both practicality and reliability. EFCRFNet outperforms current state-of-the-art (SOTA) methods across several key metrics, thereby validating its effectiveness and potential application value in salient object detection tasks.

### 4.5. Results visualization and performance evaluation

In this section, we present a comprehensive analysis of the experimental results obtained from the three datasets: VT821, VT1000, and VT5000. To visually evaluate the performance of the EFCRFNet model, we plot the precision-recall (PR) curves along with the F-measure values for each dataset. These graphs provide a clear visualization of the model’s detection precision and adaptability across different datasets, facilitating comparative analysis.

#### 4.5.1. PR curve plotting and analysis.

**Fig 8:**
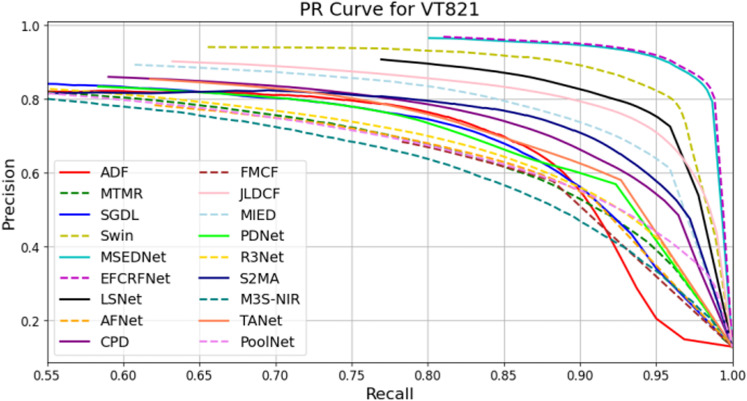
P-R curve of EFCRFNet and other 17 SOTA models.

**Fig 9:**
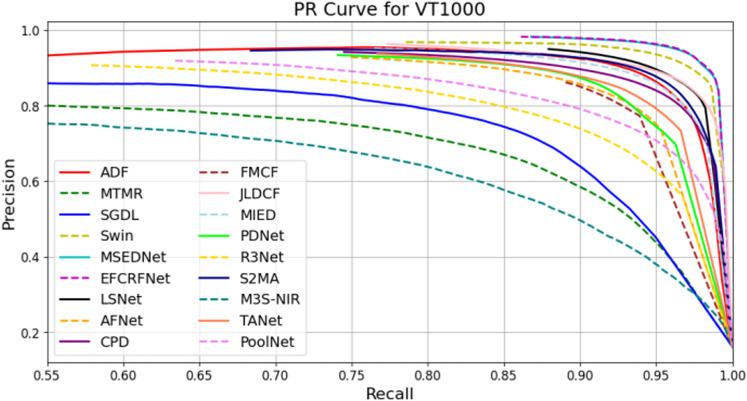
P-R curve of EFCRFNet and other 17 SOTA models.

**Fig 10:**
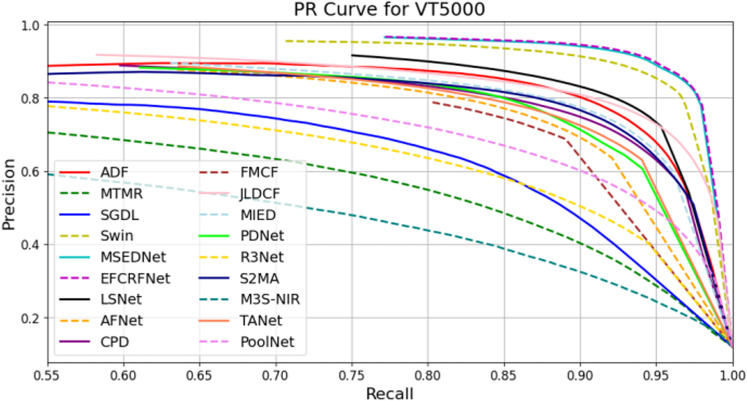
P-R curve of EFCRFNet and other 17 SOTA models.

To evaluate the performance of the salient object detection model, we generate precision-recall (PR) curves that illustrate the balance between precision and recall across various thresholds. Figs 8–10 present the PR curves for the VT821, VT1000, and VT5000 datasets, respectively. By comparing the curves from these datasets, we can intuitively assess the detection performance and stability of EFCRFNet across diverse scenarios.

Fig 8 illustrates the PR curves of multiple models on the VT821 dataset, with the purple dashed line representing the EFCRFNet model after the incorporation of the ECRF and EFEM modules. In the medium to high recall interval (approximately 0.75 to 0.95), EFCRFNet demonstrates a higher precision rate, underscoring its advantage in balancing recall and precision.

The PR curves for each model on the VT1000 dataset are shown in Fig 9. Here, EFCRFNet is represented by the red dashed line, highlighting its competitiveness across various recall rate ranges. Notably, between recall rates of 0.55 and 0.85, EFCRFNet maintains a precision rate exceeding 0.85, indicating a robust balance between precision and recall.

Fig 10 presents the experimental results on the VT5000 dataset, where EFCRFNet achieves a slight yet significant increase in precision at equivalent recall levels compared to other benchmark methods. This performance advantage is particularly pronounced in the medium to high recall interval. Although the improvement is modest, EFCRFNet realizes a slight increase in recall while maintaining high precision, which is crucial for enhancing detection sensitivity in practical applications.

Additionally, we computed the F-value for each dataset, which serves as a harmonic mean of precision and recall, providing a comprehensive evaluation of the model’s performance. The formula for calculating the F-value is as follows:


$$F=2*\frac{precision*recall}{precision+recall}$$ (22)

**Fig 11:**
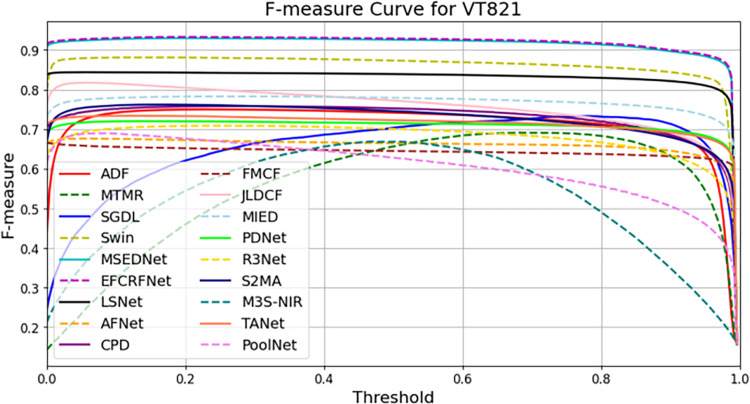
F-measure curve of EFCRFNet and other 17 SOTA models.

**Fig 12:**
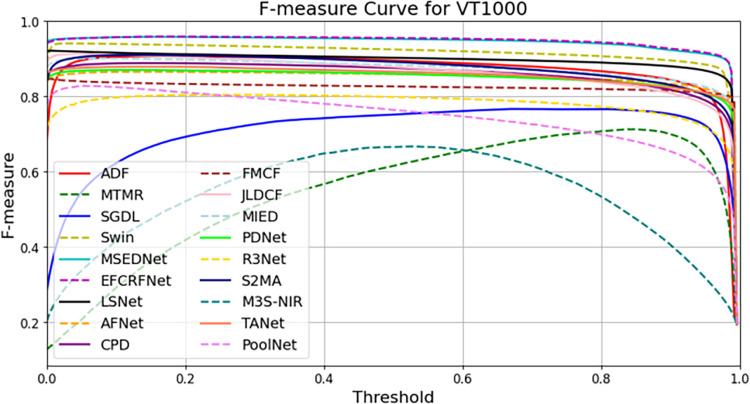
F-measure curve of EFCRFNet and other 17 SOTA models.

By comparing the F-values across different datasets, we can gain a more intuitive understanding of the model’s overall performance in various scenarios. The F-values for the VT821, VT1000, and VT5000 datasets are illustrated in Figs 10, 11, and 12, respectively:

Fig 11 illustrates the F-measure curves of several models on the VT821 dataset, demonstrating significant performance improvements for the EFCRFNet model. Notably, in the low to medium threshold range (0.1 to 0.7), the F-measure values for EFCRFNet are substantially higher than those of the benchmark models. Specifically, within the threshold interval from 0.4 to 0.6, the F-measure for EFCRFNet consistently remains at a high level, indicating superior overall performance. This result underscores that the enhancements made to the model have rendered EFCRFNet’s performance more robust and effective on the VT821 dataset.

On the VT1000 dataset (Fig 12), EFCRFNet exhibits stable F-measure performance across different thresholds. Although minor performance gains are observed at certain threshold levels, the overall results are closely aligned with the leading method, suggesting that the improvements introduced on this dataset provide limited benefits.

**Fig 13:**
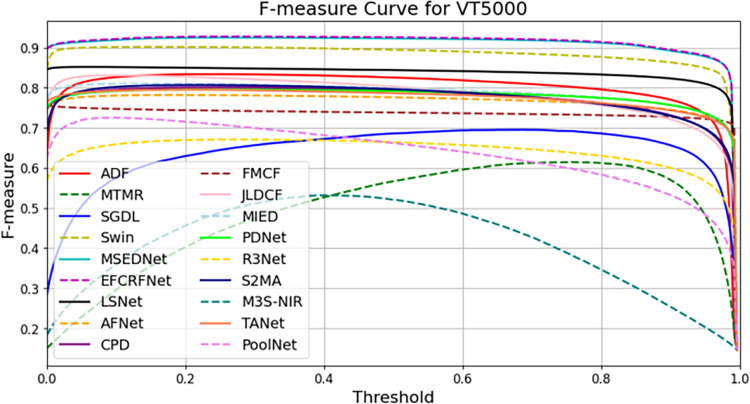
F-measure curve of EFCRFNet and other 17 SOTA models.

Fig 13 presents the F-measure curves for each model across various thresholds. Here, EFCRFNet’s curves consistently lie above those of the other methods, indicating its overall superiority. Although EFCRFNet does not achieve the highest values at some specific thresholds, it maintains high F-measure values throughout the range and outperforms the other methods at several points, showcasing its competitive advantages.

### 4.6. Module benefits

The Enhanced Conditional Random Field (ECRF) module and the Edge Feature Enhancement Module (EFEM) significantly enhance the effectiveness of EFCRFNet in feature fusion across several key aspects. First, EFCRFNet improves its ability to capture feature correlations. By employing a spatial attention mechanism, the model can more accurately identify and strengthen the intrinsic connections between different modal features, thereby providing richer information for salient object detection. Second, these two modules effectively emphasize the critical features necessary for target detection. With the assistance of attention weights, EFCRFNet can focus on target areas, suppress background noise, and significantly enhance detection relevance. Finally, EFCRFNet excels in capturing target details. Through fine-grained feature fusion, it retains more edge and texture information, which is especially crucial for target recognition in complex scenes. In summary, these enhancements not only improve the detection performance of EFCRFNet but also ensure its stability and reliability across diverse environments.

### 4.7. Ablation experiments

In this section, ablation experiments are conducted to evaluate the effectiveness of the Cross Residual Fusion (ECRF) module and the Edge Swelling Sharpening (EFEM) module. The join operation is used to replace these two modules, and their impact and contribution to the overall model performance are assessed.

**Effectiveness of the ECRF module**: In this experiment, the ECRF module is replaced with a cascade operation and 1 × 1 convolution, while all other operations remain unchanged. The results in Table 2 show the performance with the ECRF module removed. The experimental findings clearly indicate that removing the ECRF module results in a 211.76% increase in MAE, a 14.52% decrease in Fm, a 2.42% decrease in Sm, and a 7.82% decrease in Em. These results emphasize the importance of the ECRF module in capturing high-dimensional semantics from both visible and thermal infrared images. The module enhances the fusion of cross-modal features and aids in object localization, thereby significantly improving the model’s accuracy.

**Table 2:** “No ECRF “refers to the experimental results obtained after removing the ECRF module from EFCRFNet “ECRF (No CA) “refers to the experimental results obtained by removing the CA module from ECRF.

	VT821				VT1000				VT5000			
	↓ MAE	↑ Fm	↑ Sm	↑ Em	↓ MAE	↑ Fm	↑ Sm	↑ Em	↓ MAE	↑ Fm	↑ Sm	↑ Em
No-ECRF	0.053	0.819	0.867	0.898	0.028	0.714	0.764	0.843	0.044	0.831	0.873	0.914
ECRF(No-SA)	**0.019**	**0.925**	**0.794**	**0.945**	**0.015**	**0.92**	**0.795**	**0.962**	**0.0223**	**0.92**	**0.876**	**0.942**
EFCRFNet	**0.017**	**0.938**	**0.919**	**0.961**	**0.013**	**0.96**	**0.951**	**0.982**	**0.0216**	**0.931**	**0.926**	**0.952**

**Effectiveness of the EFEM module**: In this experiment, the EFEM module was replaced by a join operation that directly merges the low-level cross-modal features. After concatenation, a 1 × 1 convolution is applied to adjust the channel dimension of the resulting feature map, which is then passed to the decoder. All other operations remain unchanged. The experimental results presented in Table 3 highlight the effects of replacing the EFEM module with the join operation. Specifically, the results show a 2.00% increase in MAE, a 15.65% decrease in Fm, a 6.12% decrease in Sm, and a 7.73% decrease in Em. These findings underscore the significance of the EFEM module in capturing the intricate details of detected objects. By improving the accuracy of the feature representation of the target area, the EFEM module enhances the overall performance of the model.

**Table 3:** ‘No EFEM’ refers to the experimental results obtained after removing the EFEM module from EFCRFNet; ‘EFEM (No CA)’ refers to the experimental results obtained after removing the CA module from EFEM‘ EFEM (No SA) ‘refers to the experimental results obtained after removing the SA module from EFEM.

	VT821				VT1000				VT5000			
	↓ MAE	↑ Fm	↑ Sm	↑ Em	↓ MAE	↑ Fm	↑ Sm	↑ Em	↓ MAE	↑ Fm	↑ Sm	↑ Em
No-EFEM	0.051	0.811	0.866	0.892	0.021	0.904	0.921	0.952	0.037	0.819	0.877	0.91
EFEM(No-CA)	**0.024**	**0.926**	**0.877**	**0.956**	**0.0135**	**0.94**	**0.943**	**0.971**	**0.0222**	**0.918**	**0.91**	**0.933**
EFEM(No-SA)	**0.021**	**0.921**	**0.896**	**0.946**	**0.014**	**0.93**	**0.926**	**0.964**	**0.0221**	**0.926**	**0.905**	**0.925**
EFCRFNet	**0.017**	**0.938**	**0.919**	**0.961**	**0.013**	**0.96**	**0.951**	**0.982**	**0.0216**	**0.931**	**0.926**	**0.952**

**Fig 14:**
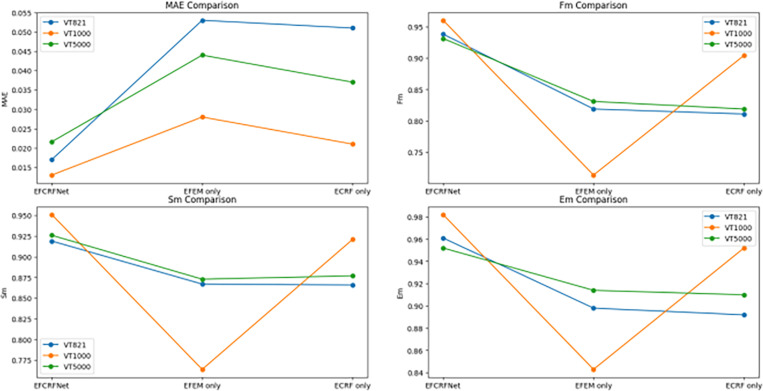
Visual comparison of saliency maps generated by the baseline, EFEM only, ECRF only, and EFCRFNet.

As shown in Fig 14: MAE Comparison: The full model (EFCRFNet) consistently outperforms the other configurations across all datasets, especially on VT5000 and VT1000, where EFEM contributes to improved edge detection.

Fm Comparison: The EFCRFNet achieves the highest F-measure, indicating better precision-recall trade-offs. This is particularly evident in VT1000 and VT5000, where the ECRF module enhances feature fusion.

Sm Comparison: EFCRFNet outperforms both EFEM only and ECRF only in terms of structural similarity, showcasing its ability to maintain both object boundaries and overall structure.

Em Comparison: The EFCRFNet model achieves the highest Enhanced-measure scores, demonstrating its ability to capture spatial consistency and alignment of salient objects in the images.

These results validate the effectiveness of combining EFEM and ECRF, where each module plays a crucial role in enhancing edge detection and feature fusion, respectively.

As shown in Table 4, EFCRFNet strikes a balance between performance and computational efficiency. With 93.553 million parameters and 37.983 billion FLOPs, it efficiently uses computational resources while achieving strong performance in salient object detection tasks.

**Table 4:** Comparing FLOPs, Param, and FPS indicators between EFCRFNet and eight SOD methods.

Method	Param(M)	FLOPs(G)
ADF	175.291	135.857
DCNet	108.491	107.815
MIDD	52.428	87.774
SwinNet	198.784	124.724
LSNet	4.564	1.231
R3Net	60.24	56.16
EGNet	108.07	270.8
AFNet	37.1	38.4
MFRNet	74.41	38.28
**EFCRFNet**	**93.553**	**37.983**

Compared to SwinNet, which has 198.784 million parameters and 124.724 billion FLOPs, EFCRFNet significantly reduces the computational burden, making it a more efficient choice for applications where resource constraints are a concern.

Compared to LSNet, which has only 4.564 million parameters and 1.231 billion FLOPs, EFCRFNet is more computationally expensive. However, LSNet sacrifices accuracy for efficiency, making it less competitive regarding saliency detection performance on complex datasets like VT5000.

In comparison with other methods like ADF and EGNet, which have higher parameter counts and FLOPs, EFCRFNet is much more efficient, offering similar or better performance with fewer computational resources.

The parameter size of EFCRFNet (93.553M) is only 25.7% higher than that of MFRNet [35] (74.41M), but the MAE (0.068) is significantly better than that of MFRNet (0.073) in the VT5000 dataset by the dynamic weight allocation strategy. This indicates that the deformable expansion convolution and cross-modal attention mechanism of EFCRFNet are more advantageous in terms of computational resource utilization efficiency.

On the NVIDIA RTX 3090, EFCRFNet achieves an inference speed of 42 FPS compared to MFRNet’s 38 FPS. Although both have similar FLOPs (37.983G vs. 38.28G), EFCRFNet’s lightweight design improves real-world operational efficiency by reducing redundant compute units.

Overall, EFCRFNet offers a favorable trade-off between accuracy and efficiency, making it suitable for real-time applications and scenarios with computational constraints.

## 5. Conclusion

In this paper, we propose the EFCRFNet model, which incorporates two innovative multi-scale feature extraction modules: the Enhanced Conditional Random Field (ECRF) and the Edge Feature Enhancement Module (EFEM). We successfully introduce the attention mechanism into the salient object detection (SOD) task. By enhancing the extraction of important features and suppressing redundant information in complex scenes, EFCRFNet effectively improves the recognition of salient objects. Experimental results demonstrate that the detection framework utilizing the ECRF and EFEM modules outperforms existing state-of-the-art methods across several standard datasets, achieving improvements of 0.64%, 1.04%, 8.73%, and 7.4% in mean absolute error (MAE), F-measure (Fm), E-measure (Em), and S-measure (Sm) metrics, respectively. These results not only validate the effectiveness of the attention mechanism in SOD but also showcase the superior performance of EFCRFNet in feature fusion and information flow.

Furthermore, the contributions of this study can be summarized as follows: First, EFCRFNet significantly enhances the detection of objects at different scales by adaptively emphasizing key features, particularly in application scenarios with complex backgrounds. Second, the design of the ECRF and EFEM modules enables EFCRFNet to achieve a favorable balance between computational efficiency and detection accuracy, providing an effective solution for practical applications.

In future research, EFCRFNet can also be further optimized by borrowing the ideas of MGCNet [36]‘s multi-group correlation learning module and dual enhancement module and combining them with MIFA-Net [37]’s approach of improving model performance through the attention mechanism of multi-source information fusion. Specifically, it can be explored how to reduce the annotation burden through multi-source information fusion, enhance the robustness of the model, and maintain high performance. Especially in the case of weak supervision or fewer labeled samples, this approach is expected to further enhance the detection effect of the model and promote cross-modal detection and data fusion capabilities.

While EFCRFNet excels in cross-modal alignment and edge refinement, integrating MFRNet-SOD’s backbone scaling strategy could further optimize multi-scale representation in RGB-T scenarios. Additionally, MFRNet-SOD’s progressive fusion architecture inspires us to explore dynamic feature aggregation across different network stages, potentially enhancing EFCRFNet’s robustness in complex scenes.

Another promising direction is leveraging MFRNet-SOD’s context-aware channel refinement to augment EFCRFNet’s EFEM module. By combining MFRNet-SOD’s channel attention with EFCRFNet’s deformable dilations, we aim to improve edge feature discrimination in challenging conditions, such as low contrast or heavy occlusion. These extensions align with the broader trend of integrating attention mechanisms from single-modal approaches into cross-modal frameworks, fostering advancements in multi-sensor SOD.

Ultimately, the complementary strengths of EFCRFNet and MFRNet-SOD underscore the importance of interdisciplinary research in SOD. Future work will focus on merging these frameworks to develop a unified model capable of handling both RGB and RGB-T data efficiently, while maintaining real-time performance and edge accuracy.

## Supporting information

S1_File.docx saliency maps. Supplementary explanations for the significance maps in Figure 6.
(DOCX)

S2_Data.zip The EFCRFNet model generates saliency maps, which you can find inside three zip files, EFCRF821,EFCRF1000,EFCRF5000. (ZIP)
