# Supplementary figures and images for: EFCRFNet: A novel multi-scale framework for salient object detection
Source: PLoS One. 2025 May 22;20(5):e0323757. doi: 10.1371/journal.pone.0323757 (PMC12097717; doi:10.1371/journal.pone.0323757)

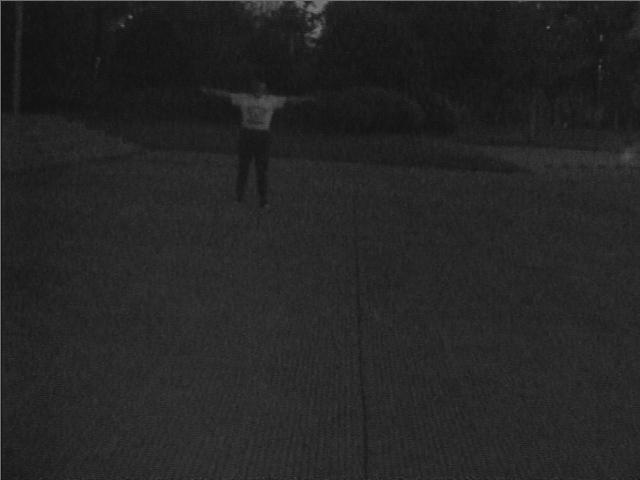

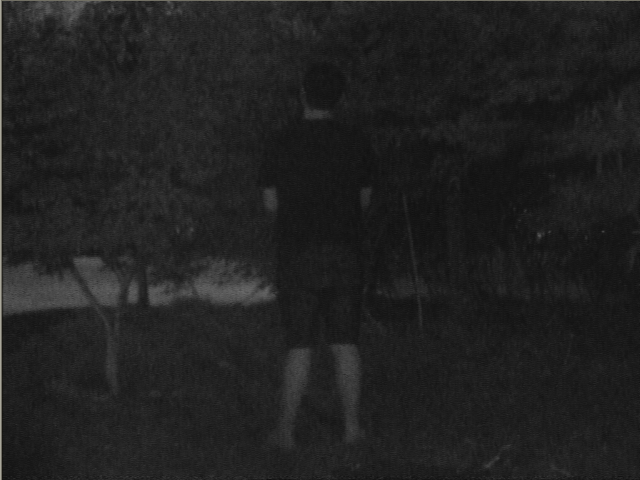

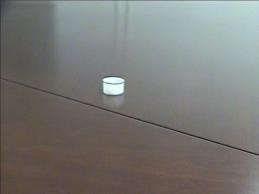

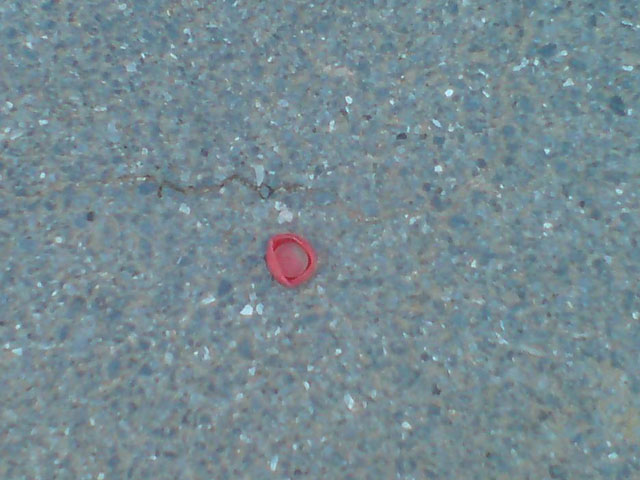


RGB


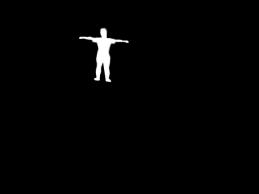

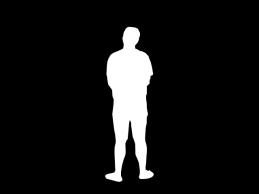

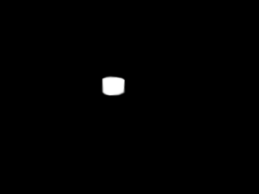

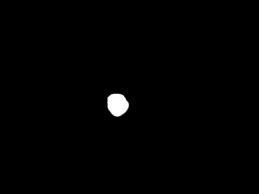


GT


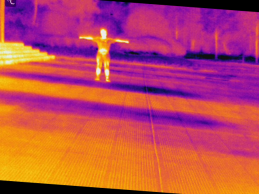

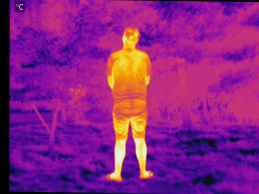

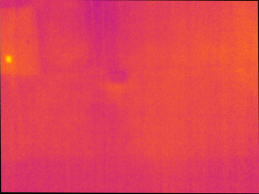

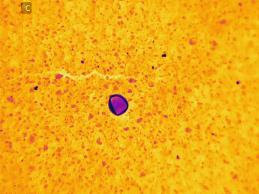


T


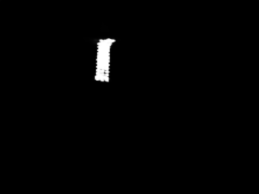

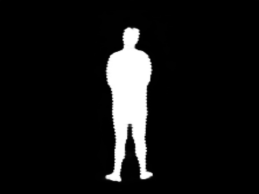

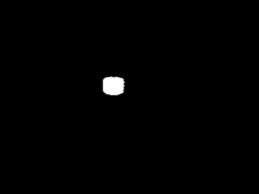

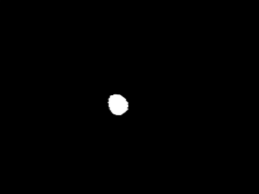


EFCRFNet


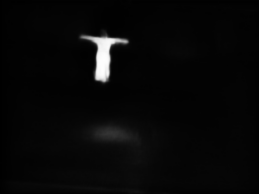

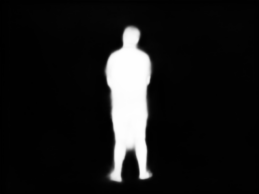

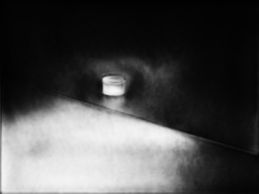

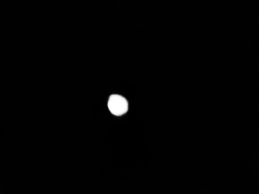


ADF


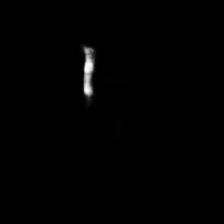

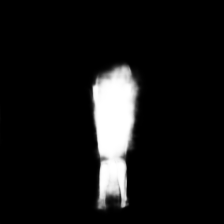

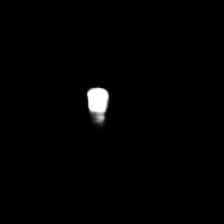

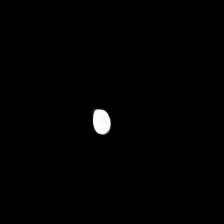


AFNet


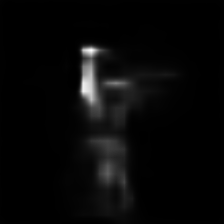

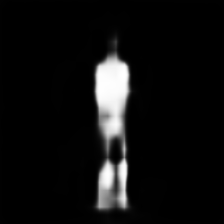

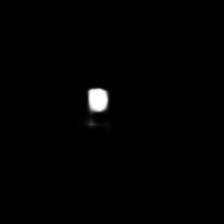

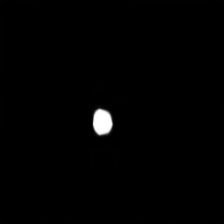


CPD


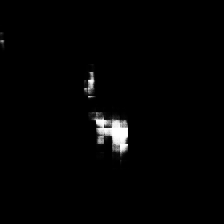

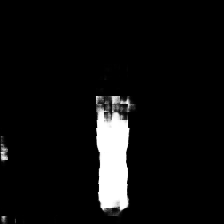

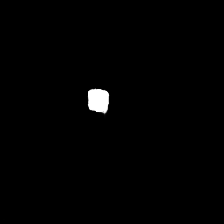

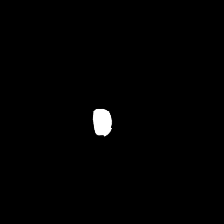


FMCF


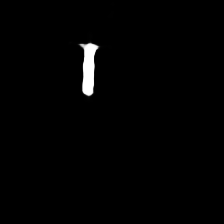

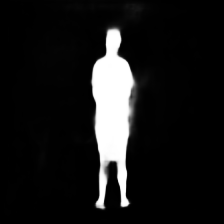

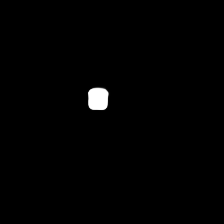

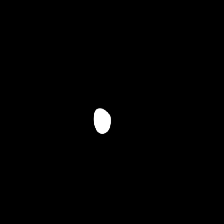


LSNet


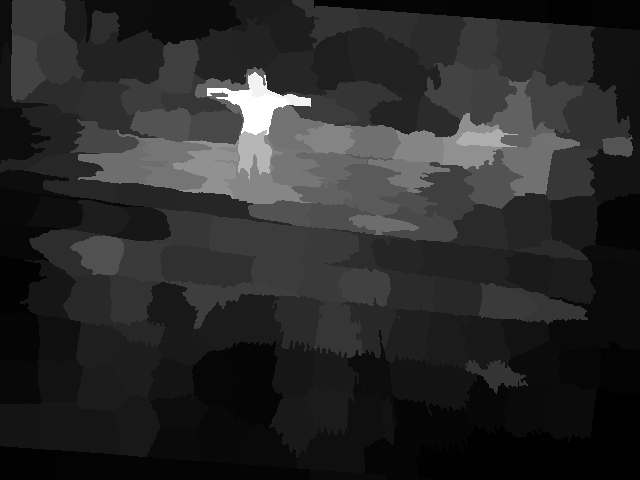

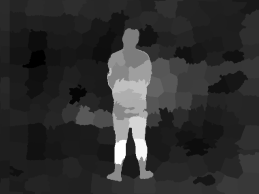

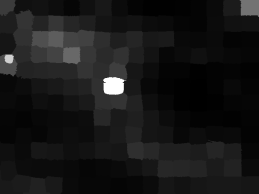

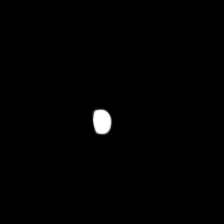


MIED


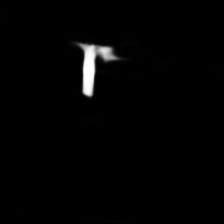

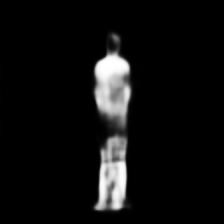

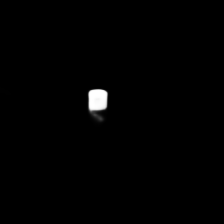

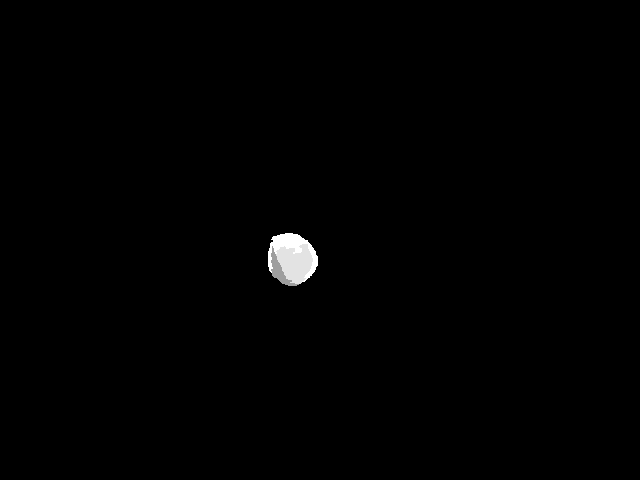


MTMR


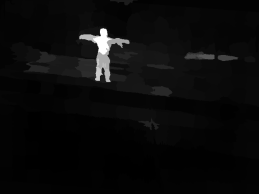

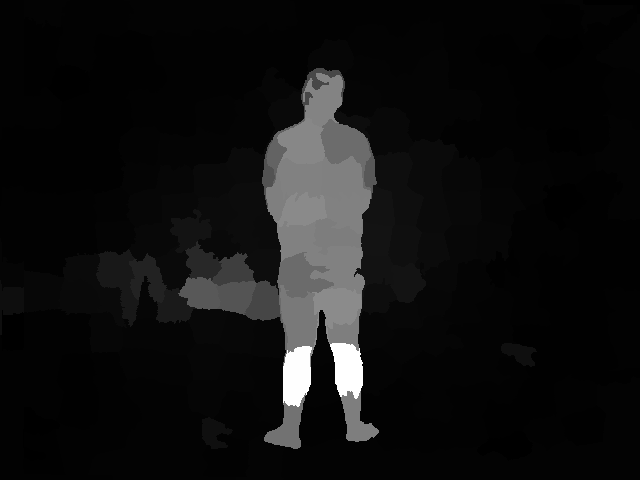

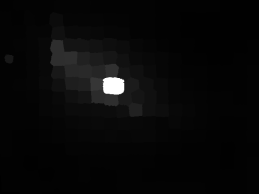

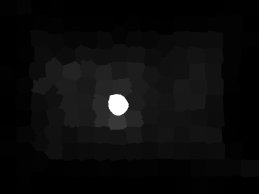


M3SNIR


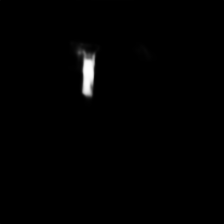

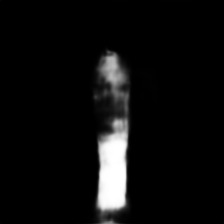

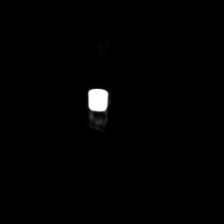

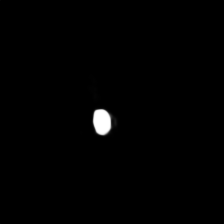


PDNet


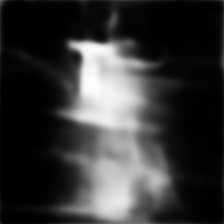

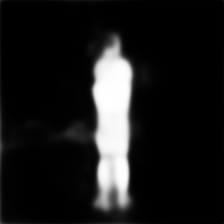

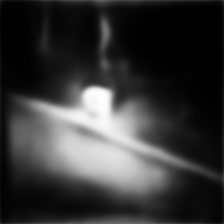

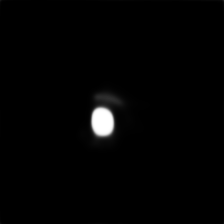


PoolNet


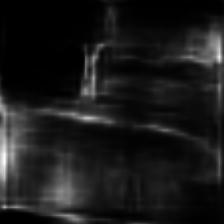

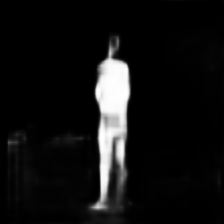

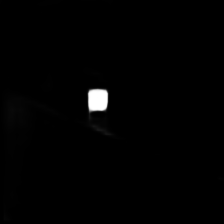

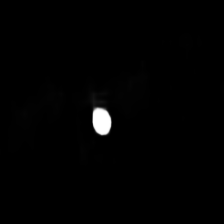


R3Net


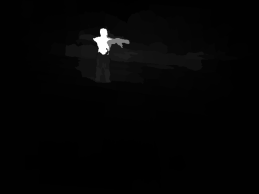

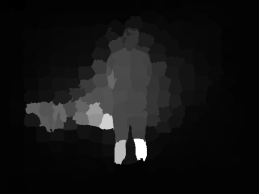

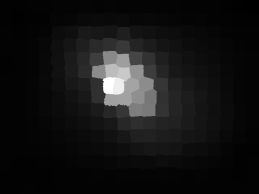

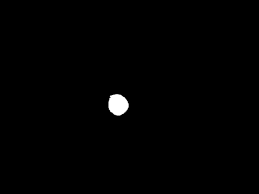


SGDL


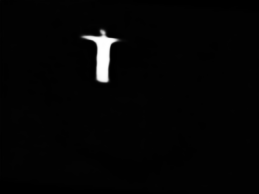

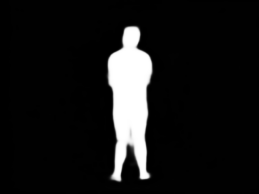

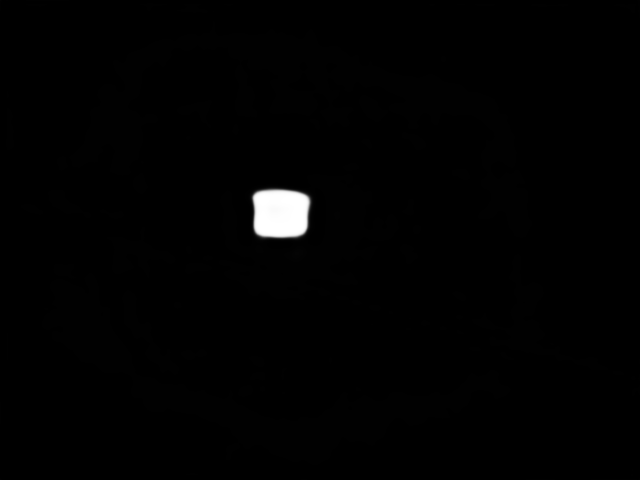

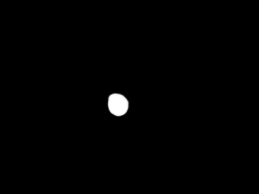


Swim


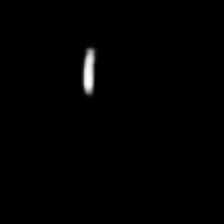

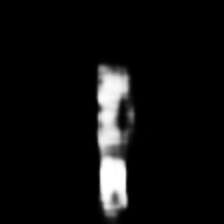

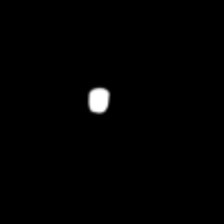

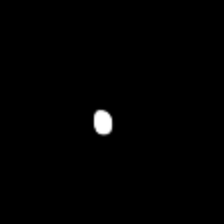


S2MA

TANet


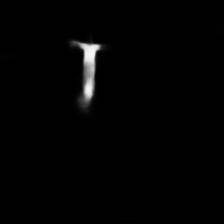

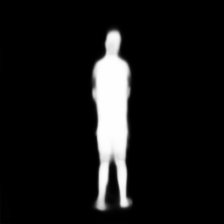

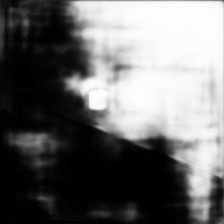

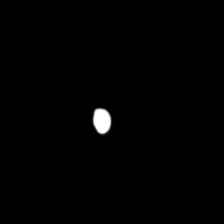

Supplement: S1_File.docx — Supplementary explanations for the significance maps in Figure 6. (DOCX) [file pone.0323757.s001.docx]
